# A single rapamycin dose protects against late-stage experimental cerebral malaria via modulation of host immunity, endothelial activation and parasite sequestration

**DOI:** 10.1186/s12936-017-2092-5

**Published:** 2017-11-09

**Authors:** Pedro Mejia, J. Humberto Treviño-Villarreal, Justin S. Reynolds, Mariana De Niz, Andrew Thompson, Matthias Marti, James R. Mitchell

**Affiliations:** 1 Department of Genetics and Complex Diseases, Harvard T. H. Chan School of Public Health, Boston, MA 02115 USA; 2 Institute of Cell Biology, University of Bern, 3012 Bern, Switzerland; 3 Wellcome Center for Molecular Parasitology, University of Glasgow, Glasgow, G12 8TA Scotland, UK; 4 Rodent Histopathology Core, Harvard Medical School, Boston, MA 02115 USA

**Keywords:** Cerebral malaria, Rapamycin, Parasite sequestration, Endothelial activation

## Abstract

**Background:**

Maladaptive immune responses during cerebral malaria (CM) result in high mortality despite opportune anti-malarial chemotherapy. Rapamycin, an FDA-approved immunomodulator, protects against experimental cerebral malaria (ECM) in mice through effects on the host. However, the potential for reduced adaptive immunity with chronic use, combined with an incomplete understanding of mechanisms underlying protection, limit translational potential as an adjunctive therapy in CM.

**Results:**

The results presented herein demonstrate that a single dose of rapamycin, provided as late as day 4 or 5 post-infection, protected mice from ECM neuropathology and death through modulation of distinct host responses to infection. Rapamycin prevented parasite cytoadherence in peripheral organs, including white adipose tissue, via reduction of CD36 expression. Rapamycin also altered the splenic immune response by reducing the number of activated T cells with migratory phenotype, while increasing local cytotoxic T cell activation. Finally, rapamycin reduced brain endothelial ICAM-1 expression concomitant with reduced brain pathology. Together, these changes potentially contributed to increased parasite elimination while reducing CD8 T cell migration to the brain.

**Conclusions:**

Rapamycin exerts pleotropic effects on host immunity, vascular activation and parasite sequestration that rescue mice from ECM, and thus support the potential clinical use of rapamycin as an adjunctive therapy in CM.

## Background

Infection with the protozoan parasite *Plasmodium falciparum* can rapidly progress into a deadly neurological syndrome known as cerebral malaria (CM), resulting in high rates of morbidity and mortality particularly in children under 5 years of age [1, 2]. Amongst infected individuals, the transition from mild malaria symptoms, including nausea and fever, to CM symptoms, including seizures and coma, is currently impossible to predict. Furthermore, no efficient treatment exists once severe symptoms arise. Therefore, it is urgent to develop novel and effective adjunctive therapies for CM.

The mechanisms leading to CM neuropathology remain poorly understood. Multiple cellular and molecular events potentially contribute independently or in combination to its aetiology. Some of these include the sequestration of infected erythrocytes in several organs including the brain; activation of vascular endothelial cells with up-regulation of adhesion molecules including ICAM-1; uncontrolled pro-inflammatory host responses to bioactive parasite products; and the activation, migration and infiltration of immune cells into inflamed tissues [3]. A better understanding of the contribution of these events to CM pathology is crucial to develop novel therapies to prevent the progression of the infection to severe disease.

The experimental cerebral malaria (ECM) model, consisting of infection of the susceptible mouse strain C57BL/6 mice with the *Plasmodium berghei* ANKA strain, mimics several aspects of the neuropathology observed in CM patients. In this model, mice suffer from recruitment of antigen-specific cytotoxic CD8+ T cells to the brain, which destroys the blood–brain barrier (BBB) in a perforin and granzyme B-dependent manner [4, 5]. In turn, disruption of brain vascular integrity results in seizures, paralysis, coma and ultimately death [6, 7]. Using the ECM model, several studies have identified modulators of host targets as potential adjunctive therapies. These include inhibition of glutamine metabolism by 6-diazo-5-oxo-l-norleucine (DON) [8], activation of the nuclear hormone receptor peroxisome proliferator activator gamma (PPAR-ɣ) by rosiglitazone [9], and inhibition of the nutrient/energy sensor mechanistic target of rapamycin complex 1 (mTORC1) kinase by rapamycin [10, 11].

Rapamycin is particularly interesting due to its known safety profiles in humans. Rapamycin (sirolimus/rapamune), a partial allosteric inhibitor of mTORC1 kinase activity, is FDA approved for use as an immunosuppressant to prevent organ transplant rejection. In the context of ECM, acute prophylactic treatment with rapamycin during the first 3 days of infection protects mice from ECM neuropathology [10, 11]. This protection occurs without affecting peripheral parasite growth, but rather via induction of activated T cells in the spleen that reduce parasite burden, while preventing pathologic migration of activated T cells to the brain [11]. Chronic rapamycin treatment beginning on day 1 or day 4 of infection also protects from ECM, but with the caveat of increasing peripheral parasitaemia and increasing proinflammatory cytokines, all suggestive of host immune alteration [10]. Based on these observations, it appears that factors inherent to timing and dosage determine the relative effect of rapamycin on the host immune response to parasite infection. Understanding this effect is key to enable clinical translation of rapamycin for CM treatment. The purpose of this study was to investigate a rapamycin dosing strategy that maximizes protection from ECM after the emergence of symptoms, but without compromising adaptive immunity. The results presented herein demonstrate that a single rapamycin dose, provided as late as day 5 of infection, protected mice from ECM neuropathology via modulation of parasite sequestration in peripheral organs, activation of splenic immunity, and prevention of neurovascular activation and BBB destruction.

## Methods

### Mice

Wild-type female C57BL/6J mice 8–10 weeks of age were purchased from Jackson Labs (Bar Harbor, ME). Animals were housed 4–5 per cage and kept under standard laboratory conditions and allowed free access to water and food.

### Ethics statement

All animal experiments were performed either with the approval of the Animal Research Ethics Committee of the Canton Bern, Switzerland and the University of Bern Animal Care and Use Committee, or the Harvard Medical Area Animal Care and Use Committee according to the PHS Policy on Humane Care and Use of Laboratory Animals by Awardee Institutions and NIH Principles for the Utilization and Care of Vertebrate Animals Used in Testing, Research and Training.

### Food

Mice were fed a purified diet (D124570B, Research Diets, New Brunswick, NJ) with 72% calories from carbohydrate (sucrose, maltodextrin, corn starch), 18% calories from protein (casein) and 10% from fat (soybean oil, lard) supplemented with 15 mg of PABA per 100 g of food. Powder diets were mixed with 1 L/kg diet of hot water containing 2% agar; the cooled mixture was given daily to mice either on an ad libitum basis.

### Malaria infection

Cryopreserved transgenic *P. berghei* ANKA parasites expressing luciferase/GFP under a constitutive promoter [12] were thawed and passaged once in vivo before being used to infect experimental mice with 0.5 million parasitized RBCs/mouse by tail vein injection. Parasites from peripheral blood were stained with SYTO16 and the percentage of infected cells was calculated by flow cytometry [13]. Individual mouse weights and food intake per cage were calculated daily.

### Luciferase-based parasite accumulation/sequestration assay

Infected mice were sacrificed on the day 6 after infection and perfused with cold PBS intracardially. The organs were harvested, weighed and homogenized in an equal volume/mg tissue of luciferase activity assay buffer (Invitrogen). Equal volumes were mixed with luciferin substrate and measured in a 96 well luminometer (Biotek Synergy 2).

### Brain vascular permeability

On day 6 after infection, mice were injected intravenously with 200 μL of PBS-2% Evans blue, sacrificed 1 h later, and perfused intracardially with cold PBS. Brains were harvested, photographed, weighed and placed in 2 mL 100% formamide (Merck) for 48 h at 37 °C. Absorbance of the formamide supernatant was measured at 620 nm. Evans blue concentration was calculated from a standard curve and results expressed as microgram of Evans blue/gram of brain tissue.

### Intra-vital microscopy

Intra-vital and ex vivo imaging were performed in 5–6 week-old female C57BL/6 mice infected with *P. berghei* ANKA expressing mCherry under the Hsp70 promoter, and Firefly luciferase under the ef1a promoter [14]. A mixture of anaesthetics comprising 125 mg/kg ketamine (Ketasol, Graeub) and 12.5 mg/kg xylazine (Xylason, Graeub), was prepared and diluted in 1 × PBS (1:2:5). Mice were injected intraperitoneally with 100 mL per 20 g of body weight, of the mixed anaesthetics. Following anaesthesia, mice were injected intravenously with 200 µL of 70 kDa fluorescin isothiocyanate (FITC)-conjugated dextran (Sigma Aldrich, St. Louis, MO) diluted in 1 × PBS, at a final concentration of 10 mg/mL. Mice were imaged immediately after FITC-Dextan injection each day post-infection, in a Leica SP8-STED confocal microscope, using a HC PL APAO CS2 63X 1.4NA oil immersion objective. In the LeicaSP8-STED microscope, a white laser was used, and wavelengths were defined for the spectra corresponding to FITC and mCherry. Experiments were performed in three mice at each day post-infection. The images were assembled and processed using Fiji imaging software.

### Isolation and analysis of leukocyte populations

Brains and spleens were harvested at the indicated days after infection following perfusion with cold PBS. Spleen single-cell suspensions were prepared by homogenization through a 70 µm cell strainer (BD Falcon). Brains were digested with 50 U/mL of type II and IV collagenase (Gibco, Invitrogen) in RPMI, and mononuclear cells isolated on a 33% Percoll density gradient (GE Healthcare). After lysing red blood cells and determining the total cell concentration, cells were incubated with an FcR blocker (Miltenyi Biotech), labelled for the extracellular antigens CD3, CD8, CD4 (BD Pharmingen), CD69, LFA-1, CD62L, CXCR3, CD25 (all from Biolegend), fixed with 4% paraformaldehyde (PFA) and stained for the intracellular markers Granzyme B (Biolegend) and Foxp3 (eBioscience) using the Cytofix/Cytoperm kit (BD Biosciences) following the manufacturer’s instructions. Single fluorochrome-labelled cells were used to compensate for spectral overlap. Isotype-matched fluorescence minus one controls were used to set gate cutoffs. Acquisition was performed using a BD Fortessa and data analysed using FlowJo (Tree Star Inc.).

### Histology

Mice were perfused intracardially with PBS on the indicated day after infection. For morphometric analyses, brains were fixed in 4% paraformaldehyde in PBS, embedded in paraffin, cut and H&E stained using standard procedures. For immunofluorescence microscopy, brains were flash frozen in liquid nitrogen and stored at − 80 °C until use. Sections were cut with a cryotome, fixed in ice-cold 4% PFA, permeabilized in 0.25% Triton X-100, washed in PBS and blocked in 10% normal goat serum diluted in TBS-T (TBS buffer containing 0.2% Tween 20). After blocking, sections were incubated with primary rabbit anti CD31 and rat anti-ICAM-1 (both from Abcam, Cambridge, MA) overnight at 4 °C. After washing with PBS, sections were labelled with Alexa 488 goat and rabbit IgG and Alexa 568 goat anti rat IgG for 2 h at room temperature. After extensive washing, slides were mounted in anti-fade mounting media with DAPI (Vector Labs, Burlingame, CA). Images were taken with a Zeiss Axio observed fluorescence microscope with an APOTOME attachment with a 20 ×/0.8 M27 plan-apochromat objective and an AxioCam monochromatic MRm digital camera. Slices stained only with secondary antibodies were used to set parameters. For quantitation, all images in both groups were taken using the same acquisition parameter. Image processing analysis was performed using the using the AxioVision 4.7.1 software as well as Fiji imaging software.

### Quantitative real-time PCR

Total RNA was extracted from frozen tissue with RNA Bee (Qiagen). Hexamer-primed complementary DNA (cDNA) was synthesized with Verso cDNA kit (Thermo Scientific) according to manufacturer’s instructions. Quantitative real-time PCR was performed with a MyIQ (Bio-Rad) using SYBR Green. Relative expression was calculated with the ΔΔC_t_ method. cDNA expression of each sample was standardized to RPLA13. Each sample was tested in duplicate at least twice. Primer sequences used: **CCL-5**, fwd: 5′-GCAAGTGCTCCAATCTTGCA-3′; rev: 5′-CTTCTCTGGGTTGGCACACA-3′. **CXCL-9**, fwd: 5′-GCCATGAAGTCCGCTGTTCT-3′; rev: 5′-GGGTTCCTCGAACTCCACACT-3′. **CXCL-10**, fwd: 5′-GACGGTCCGCTGCAACTG-3′; rev: 5′-GCTTCCCTATGGCCCTCATT-3′. **ICAM-1**, fwd: 5′-GCCTCCGGACTTTCGATCTT-3′; rev: 5′-GTCAGGGGTGTCGAGCTTTG-3′. **Pb18S**, fwd: 5′-AAGCATTAAATAAAGCGAATACATCCTTAC-3′; rev: 5′-GGAGATTGGTTTTGACGTTTATGTG-3′. **P-Selectin**, fwd: 5′ CCCTGGCAACAGCCTTCAG-3′; rev: 5′-GGGTCCTCAAAATCGTCATCC-3′. **VCAM**, fwr: 5′-AGTTGGGGATTCGGTTGTTCT-3′; rev: 5′-CCCCTCATTCCTTACCACCC-3′.

### Immunoblots

Snap frozen tissues were homogenized in 1 mL of lysis buffer consisting of 10% glycerol, 1% NP-40, 1 mM MgCl_2_, 150 mM NaCl, 50 mM β-glycerophosphate, 20 mM Tris–HCl pH 7.4 supplemented with a protease inhibitor and phosphatase inhibitor cocktails (Thermo Fisher Scientific, Rockford, IL) with an IKA T10 Ultra-Turrax (Wilmington, NC) tissue disperser. Homogenized samples were centrifuged for 20 min at 10,000*g*, and the protein concentration determined from extracts using the Pierce BCA protein assay kit (Thermo Scientific). 40 μg of protein extract was resolved by SDS-PAGE, transferred to Immobilon-P polyvinylidene difluoride (PDVF) membranes (Millipore), blocked in 5% skim milk diluted in TBST (10 mM Tris–HCl pH 7.4 plus 0.1% Tween 20), washed in TBST, and incubated overnight at 4 °C with primary antibody (CD36 and tubulin, both from Abcam, Cambridge MA) 1:1000 diluted in antibody buffer (5% BSA, 0.1% sodium azide in TBST). After washing in TBST, membranes were incubated 90 min at room temperature with goat anti-mouse or goat anti-rabbit IgG conjugated with horseradish peroxidase (Dako, Carpinteria, CA) diluted 1:5000 in 5% skim milk. After extensive washing with TBST, blots were developed using the Super Signal West Femto chemiluminescent substrate kit (Pierce, Thermo Scientific).

### In vivo rapamycin treatments

A stock solution of rapamycin (LC Laboratories, Woburn MA) dissolved in 100% ethanol was further diluted in 5% Tween/5% PEG-400 vehicle solution and was administered i.p. to infected mice at 5 or 25 mg/kg/day on day 4, 5 or 6 of infection; vehicle solution was used as a control.

### Statistics

Data are expressed as mean ± SEM unless indicated otherwise. Statistical analyses were performed in GraphPad Prism with Mann–Whitney, one-way analysis of variance (ANOVA) or Kaplan–Meier survival tests as indicated.

## Results

### A single dose of rapamycin rescues mice from late-stage ECM

**Fig. 1:**
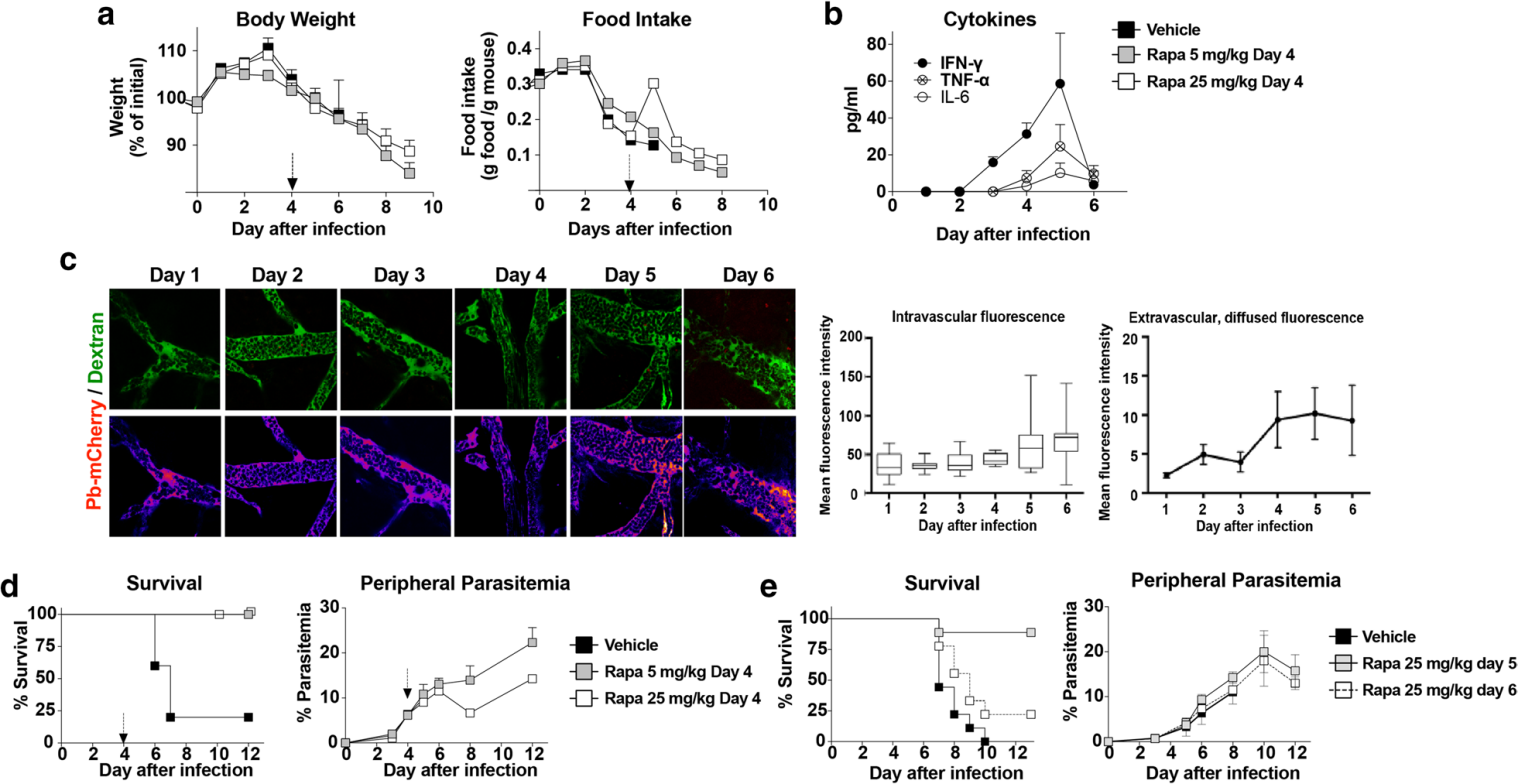
A single dose of rapamycin rescues mice from late-stage ECM. **a** Body weights and food intake of female C57BL6 mice infected with *Plasmodium berghei* ANKA on day 0 and injected with vehicle or rapamycin (5 or 25 mg/kg) on day 4 (arrow) post-infection as indicated. **b** Circulating cytokines over the course of infection in female C57BL6 mice infected with *P. berghei* ANKA on day 0 (n = 4/time point). **c** Time course of infection-induced brain vasculature permeability. C57BL6 mice (3 per day) were infected with *P. berghei* ANKA that expresses mCherry (Pb-mCherry). On the indicated day, mice were injected with FITC-Dextran to analyze intravascular diffusion and extracellular permeability using intra-vital microscopy. Upper panels are composite images showing FITC-mCherry expression. The lower panels are representative images of the FITC channel, displayed as heat-map LUTs for quantitation. Evaluation of intravascular and extravascular—diffused-FITC-dextran mean fluorescence intensity (MFI) is quantified and plotted at right. **d** Survival and peripheral parasitaemia in female C57BL6 mice infected on day 0 and injected with vehicle or rapamycin (5 or 25 mg/kg) on day 4 (arrow) post-infection as indicated (n = 5/group). **e** Survival (n = 9/group) and peripheral parasitaemia (n = 4/group) of mice infected on day 0 and treated on day 5 or 6 post-infection with vehicle alone or with rapamycin (25 mg/kg) as indicated. Values are mean ± SEM

Previous studies have demonstrated that early rapamycin treatment on days 1–3 post-infection with *P. berghei* ANKA, before onset of symptoms, protects mice against ECM pathology and mortality [11]. Here, the efficacy of a single injection of vehicle or rapamycin at different doses (5 or 25 mg/kg), provided on day 4 post-infection when symptoms including weight loss and reduced food intake indicative of sickness behaviour emerge (Fig. 1a), was tested. At this time point, an increase in circulating pro-inflammatory cytokines likely causative of this anorectic response, including IFN-γ IL-6 and TNFα, was observed (Fig. 1b). Also on day 4, increased brain vascular leakage was observed by intra-vital confocal microscopy, evidence of initial blood brain barrier dysfunction (BBB) (Fig. 1c). Vascular leakage remained elevated on days 5 and 6 post infection (Fig. 1c).

Clinically, both the high and low doses of rapamycin resulted in complete abrogation of neurological symptoms, and protected against the high mortality associated with this model (Fig. 1d). Although chronic mTORC1 inhibition can interfere with host immune clearance of parasites [10] or inhibit parasite growth directly [15], a single rapamycin dose did neither, as peripheral parasitaemia levels, compared to vehicle treated controls, were not significantly affected (Fig. 1d). Interestingly, infection-induced anorexia was transiently but significantly abrogated on the day after rapamycin treatment, but only at the highest dose (Fig. 1b). The efficacy of treatment with rapamycin at a later time point (days 5 and 6), when brain vascular dysfunction is more pronounced and better established, was next tested. As shown in (Fig. 1e), mice treated with a single high dose (25 mg/kg) of rapamycin as late as day 5 post infection were also protected from neuropathology and death. However, treatment at day 6 only conferred a non-significant 25% survival rate (Fig. 1e). Nonetheless, neither day 5 nor day 6 rapamycin treatment affected the course of peripheral parasitaemia (Fig. 1e). Taken together, the results show that a single dose of rapamycin, provided as late as day 5 of infection, after the emergence of sickness behaviour and vascular leakage, protected mice from ECM. In order to establish the mechanism(s) involved in the protection afforded by rapamycin, subsequent experiments were performed using a dosing strategy consisting of one injection of 5 mg/kg on day 4 post-infection, which maximizes protection from ECM after sickness behavior and vascular leakage associated with ECM have emerged.

### Rapamycin inhibits parasite sequestration in peripheral organs

**Fig. 2:**
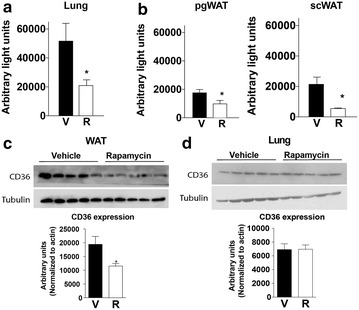
Rapamycin treatment inhibits parasite sequestration. C57BL6 mice were infected with transgenic luciferase-expressing *P. berghei* ANKA on day 0, and treated with 5 mg/kg of rapamycin (R) or vehicle (V) on day 4 post infection. Luciferase activity was measured in perfused tissues at day 6. **a** Ex vivo luciferase activity indicative of parasite sequestration in lung (**a**) and white adipose tissue (**b**); perigonadal, PgWAT; subcutaneous, ScWAT). **c**, **d** Immunoblot and quantification of CD36 protein expression in PgWAT (**c**) and lung (**d**) of day 6 infected mice treated on day 4 with vehicle or rapamycin (5 mg/kg). Data are mean ± SEM; n = 5/group

Cytoadherence, or sequestration of parasitized red blood cells (RBSs) to endothelial cells in peripheral organs including white adipose tissue (WAT) and lungs constitutes a mechanism by which mature *Plasmodium* parasites avoid circulation through and clearance by the spleen [4]. To monitor sequestration, a *P. berghei* ANKA strain constitutively expressing a GFP-luciferase transgene was employed [12], and used to measure luminescence in extracts of perfused tissues on day 6 post-infection. The results demonstrated that a single rapamycin treatment on day 4 post infection significantly reduced sequestration in lung (Fig. 2a) and WAT, including both perigonadal and subcutaneous depots, relative to vehicle-treated controls (Fig. 2b).

The potential mechanisms by which rapamycin reduced peripheral sequestration were next explored. In mice and humans, the fatty acid receptor CD36 is a major host determinant of sequestration in peripheral tissues including lung and WAT (but not brain) [16–19]. Interestingly, rapamycin treatment resulted in reduced CD36 protein expression in WAT but not lungs on day 6 post-infection (Fig. 2c, d). These results suggest the existence of CD36 dependent and independent mechanisms of peripheral parasite sequestration, selectively and differentially regulated in different tissues by mTORC1 activity.

### Rapamycin inhibits T cell migration capacity and effector functions in the spleen

**Fig. 3:**
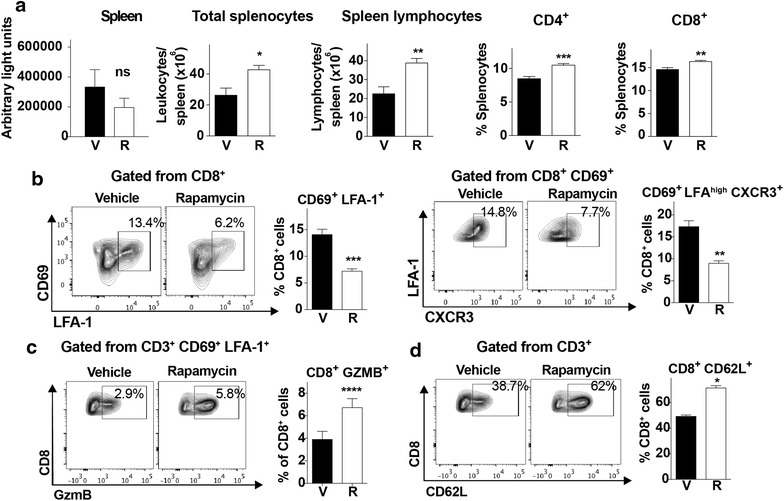
Rapamycin alters splenic lymphocyte migratory capacity and effector functions. **a** Ex vivo luciferase activity in spleen on day 6 after infection of mice with transgenic luciferase-expressing *P. berghei* ANKA treated with vehicle or rapamycin (5 mg/kg) on day 4. Total numbers of splenocytes, spleen lymphocytes and relative numbers of CD4+ and CD8+ T cells of vehicle (V) or rapamycin treated (R) mice on day 6 post infection. **b** Relative CD8+ lymphocyte numbers, with representative dot plots of activated CD8+ T cell populations expressing the indicated markers in the spleen of the indicated treatment groups on day 6 post infection. **c** Relative numbers with representative dot plots of granzyme B+ effector T cells gated from the CD3+ CD69+ LFA-1+ population. **d** Relative numbers of CD62L+ naïve CD8+ T cells gated from CD3+ splenocytes with representative dot plots from the indicated treatment group on day 6 post infection. Data are mean ± SEM; n = 5/group

Because sequestration of mature parasites in lungs and WAT represents a strategy to avoid clearance of parasitized erythrocytes in spleen, reduced sequestration (Fig. 2) without a significant increase in circulating parasitized RBCs (Fig. 1) could potentially be explained by increased splenic clearance. Parasite-derived luciferase activity detected in the spleens from mice treated with rapamycin on day 4, tended to be reduced by day 6 compared to vehicle-treated controls per mg of tissue, although this difference did not reach statistical significance (Fig. 3a). However, as splenic clearance may also destroy parasite luciferase activity, it is not a reliable measure of clearance activity.

Host immune cell-based measures of splenic activity were next explored. Treatment with rapamycin on day 4 translated into an increase in total splenocytes, as well as an increase in total lymphocytes, including both CD4+ and CD8+ T cells, by day 6 post-infection (Fig. 3a). Amongst CD8+ T cells, the proportion displaying the activated phenotype, characterized by CD69, LFA-1 and CXCR3 expression, was significantly reduced following rapamycin treatment (Fig. 3b). However, amongst the population of activated CD8+, CD69+, LFA1+ T cells, the proportion expressing granzyme was increased upon rapamycin treatment, consistent with more robust effector function, possibly leading to enhanced clearance (Fig. 3c). Lastly, the proportion of naïve CD62L+ CD8+ T cells, characterized of reduced migratory capacity, significantly increased following rapamycin treatment (Fig. 3d). Together, these data suggest that a single dose of rapamycin on day 4 of infection alters splenic T cell differentiation, resulting in an overall decrease in T cell activation and migratory capacity, but an increase in effector function.

### Rapamycin modulates the recruitment of effector T cells to the brain

**Fig. 4:**
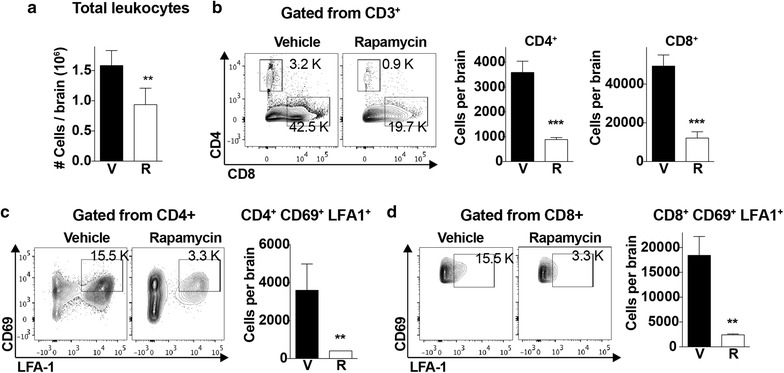
Rapamycin modulates the recruitment of effector T cells to the brain. **a** Total number of leukocytes (in thousands, K) isolated from brains of mice treated with vehicle or rapamycin (5 mg/kg) on day 4 and collected on day 6 expressed per brain. **b** Total numbers with representative dot plots of CD4+ and CD8+ T cells from brains as determined by flow cytometry. CD4+ and CD8+ populations where gated from CD3 + cells; n = 5/group. **c**, **d** Total numbers with representative dot plots of activated CD4+ (**c**) and CD8+ (**d**) T cells from mice of the indicated treatment group on day 6 post infection. Data are mean ± SEM; n = 5/group

The simultaneous presence of parasites and activated CD8+ T cells in brains of infected mice is required for BBB disruption and ECM neuropathology [4]. Therefore, the effect of day 4 rapamycin treatment on T cell accumulation in brains of infected mice by day 6 post-infection was investigated. Quantitation of leukocytes isolated from brains of infected animals after perfusion on day 6 revealed significantly fewer total leukocytes present in the rapamycin group (Fig. 4a), including reduced accumulation of CD4+ and CD8+ T cells (Fig. 4b). Moreover, these fewer CD4+ and CD8+ T cells displayed reduced expression of activation markers such as CD69 and LFA1 (Fig. 4c, d), consistent with data in spleen showing that mTORC1 inhibition of T cells on day 4 prevented T cell activation and trafficking to the brain later in infection.

### Modulation of brain vascular endothelial activation upon rapamycin treatment

**Fig. 5:**
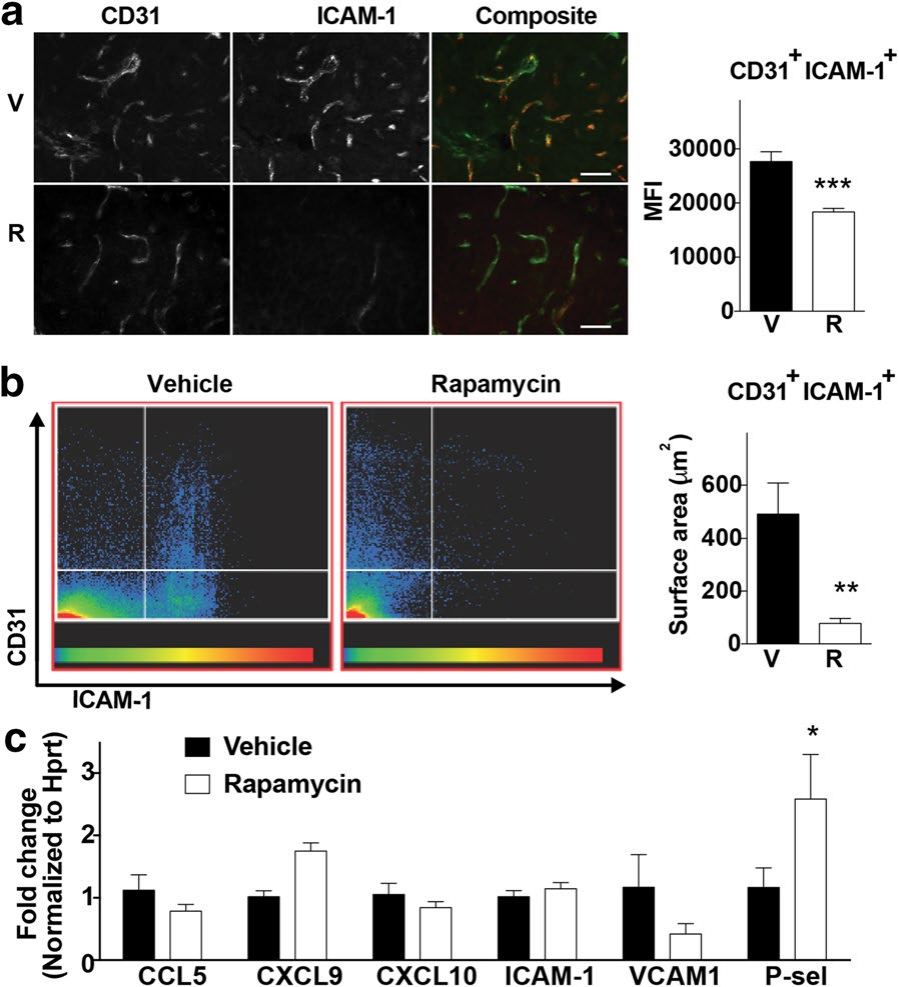
Rapamycin modulaes endothelial activation in the brain. **a** Representative immunohistochemical images of day 6 brains from vehicle or rapamycin treated mice, stained for the endothelial marker CD31 (green) and ICAM-1 (red). Bar graph next to the micrographs shows the quantitation of the ICAM-1 mean fluorescence intensity (MFI) of over 20 fields of view, each one consisting of multiple optical sections 30 μm deep. Scale bar = 50 μm. **b** Dot plots showing the distribution and surface area representing CD31/ICAM-1 co-localization in endothelial cells by microscopy. Color intensity scale represents the frequency of events within the distribution. Bar graph next to plots show the quantitation of the surface area presenting ICAM-1 co-localization on CD31 brain endothelial cells. **c** Relative gene expression of chemokines and endothelial activation markers on day 6 of infection in brains of mice (n = 5/group) treated with vehicle (V) or rapamycin (R, 5 mg/kg) on day 4. Gene expression was analyzed using the ^∆∆^CT equation, and fold change is expressed respect to the vehicle treated group. Data represent mean ± SEM

Vascular endothelial cell activation and dysfunction is another feature of CM that correlates with pathology [20]. mTORC1 plays a key role in controlling the expression of endothelial cell adhesion molecules such as ICAM-1, VCAM-1 and P-selectin [21–23]. Analysis of ICAM-1 expression in brain sections on day 6 post-infection revealed rapamycin-dependent reduction in the intensity of endothelial ICAM-1 expression (Fig. 5a) as well as the total endothelial area expressing this adhesion molecule (CD31/ICAM-1 vascular co-localization, Fig. 5b). Interestingly, except for VCAM-1, rapamycin did not reduce mRNA expression of ICAM-1 or other chemokines crucial for T cells migration into the brain (Fig. 5c). These results are consistent with effects of rapamycin on neurovascular activation on the post-transcriptional level.

### Rapamycin reduces brain pathology

**Fig. 6:**
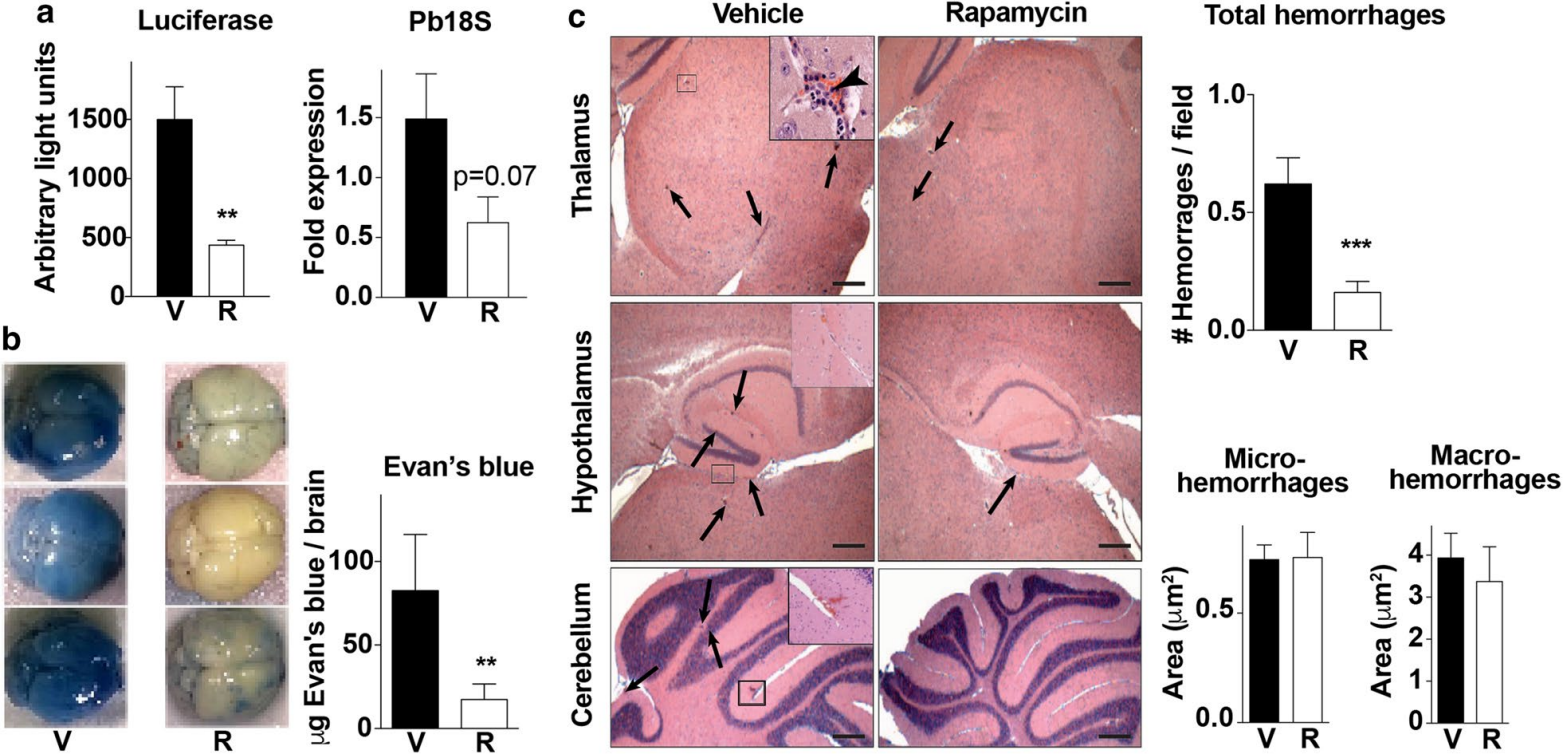
Rapamycin reduces brain pathology. **a** Luciferase activity and *Pb*18S expression indicative of luciferase-transgenic parasite accumulation in the brain of perfused mice (n = 5/group) on day 6 of infection treated on day 4 with vehicle (V) or rapamycin (R, 5 mg/kg) as indicated. **b** Quantitation of Evan’s blue dye in brains and photographs of representative brains from mice (n = 4–5/group) treated with vehicle or rapamycin (5 mg/kg) on day 4 and injected with Evan’s blue dye on day 6 after infection to assess blood–brain barrier function. **c** Representative micrographs of brains from vehicle and rapamycin treated (5 mg/kg) mice 6 days after malaria infection. Upper panels show representative images of the thalamic area, which presents disperse micro and macro-haemorrhages (arrows) scattered throughout the parenchyma. It is also evident in this image the vascular leukostasis (Inset), caused by clustering of leukocytes and infected RBCs (arrow head) within the blood vessels. Mid panels show representative images of the hypothalamus region presenting leukostasis and scattered micro-haemorrhages (arrows), defined as haemorrhagic areas smaller than 2 μm^2^ (Inset). Lower panels show representative images comparing the cerebellum of vehicle and rapamycin treated mice. Inset shows a macro-haemorrhage in the molecular layer. Macro-haemorrhages (arrows) were defined as haemorrhages exceeding 2 μm^2^. Quantitation of total number of brain haemorrhages per field of view are shown. Morphometric analysis showing the surface area of haemorrhages in whole brain sections are also shown. Hemorrhages were divided in micro-haemorrhages (< 2 μm^2^) and macro-haemorrhages (> 2 μm^2^). Data represent the mean ± SEM of over 100 fields of view. Scale bar = 100 μm. Inlets are ×40 magnified images of the selected regions

ECM neuropathology results from accumulation of infected RBCs in the brain, together with activated CD8+ T cells, which promotes destruction of the BBB [4, 24]. Having demonstrated vascular leakage starting as early as day 4 post-infection, the effect of a single dose of rapamycin at this time point on brain pathology was evaluated. Rapamycin treatment on day 4 significantly reduced the accumulation of parasites in brains on day 6 post-infection (Fig. 6a). This result was obtained by quantitating luminescence from parasites and confirmed by measurement of the parasite-specific *Pb18s* gene from brains using real-time PCR (Fig. 6a).

Reduce parasite brain sequestration correlated with preserved BBB integrity, as evidenced by reduced vascular leakage of injected Evan’s blue dye in rapamycin-treated mice (Fig. 6b). Furthermore, histological analyses revealed significantly fewer brain haemorrhages in the rapamycin-treated group compared to controls (Fig. 6c). However, no differences in the size of the haemorrhages were observed (Fig. 6c).

## Discussion

**Fig. 7:**
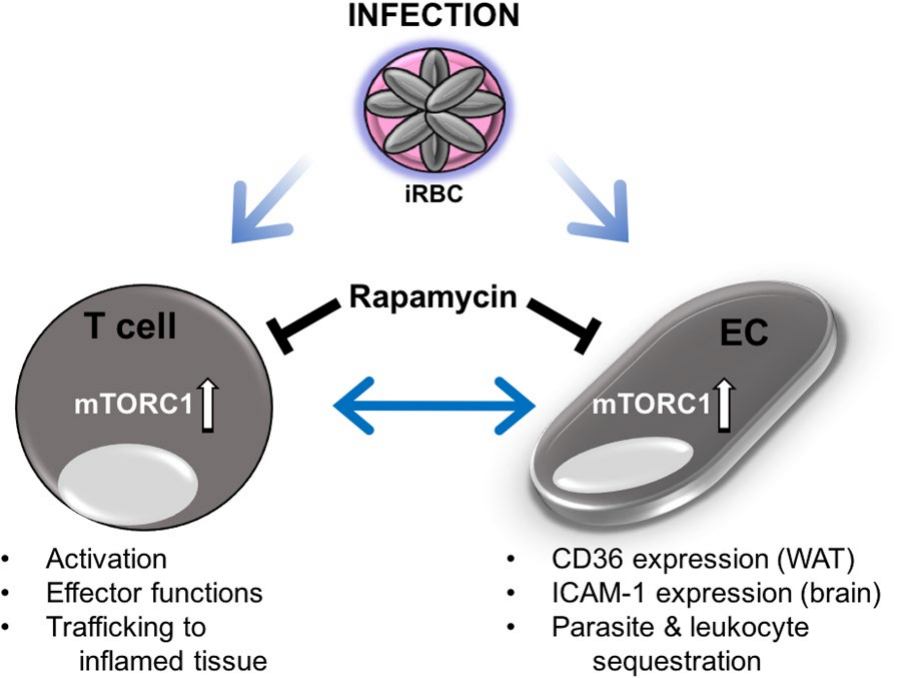
Model of pleiotropic actions of rapamycin against ECM. Rapamycin treatment targets multiple tissues and cell types during infection and results in protection against neuropathology and mortality in the ECM model. Consistent with its role as a master regulator of different key activities in multiple cell types, inhibition of mTORC1 activity with rapamycin during infection decreases activation and inflammatory migration of T cells, and endothelial cell (EC) expression of adhesion molecules. Importantly, rapamycin treatment modulates CD36 expression in specific tissues including WAT resulting in reduced parasite sequestration. Furthermore, decreased T cell activation and migration via mTORC1 inhibition reduces expression of adhesion molecules by EC which in turn decreases migration and adhesion of T cells to inflamed tissues

This study demonstrates for the first time that mTORC1 inhibition with rapamycin can offer significant protection against ECM neuropathology even with a single dose as late as day 5 post-infection. Consistent with the pleiotropic function of its target, mTORC1, protection by rapamycin treatment was afforded through inhibition of multiple pathogenic mechanisms. These included: parasite accumulation in peripheral organs in part via decreased CD36 expression; splenic CD4+ and CD8+ T cell activation and trafficking to the brain; and activation of neurovascular endothelium and BBB breakdown. A schematic model of the pleiotropic effects of rapamycin during infection that result in protection against ECM is provided in Fig. 7.

mTORC1 plays a central role in activation and trafficking of antigen-stimulated CD4+ and CD8+ T cells. mTORC1 activation prevents homing of naïve and memory T cells to secondary lymph nodes and instead redirects them to sites of inflammation [25]. This occurs through differential regulation of the transcription factors KLF2 and T-bet [26]. Repression of KLF2 expression by mTORC1 reduces expression of its targets CCR7, CD62L and sphingosine-1 phosphate receptor 1 (S1PR1) on naïve and memory T cells [26]. At the same time, activation of T-bet by mTORC1 increases expression of CXCR3 and P-selectin ligands that coordinate the migration of T cells towards sites of inflammation. T-bet dependent gene expression also promotes the expression of CD8+ effector molecules in T cells [27, 28]. Interestingly, S1P signaling and T-bet expression have both been implicated in the pathology of human and experimental CM [29, 30]. In this context, clinical studies have shown increased predisposition to CM development in patients bearing low SP1, while increased bioavailability via pharmacological means improves survival and vascular integrity in the ECM model [29]. Furthermore, T-bet ablation is associated with protection from ECM [30].

The results presented in this study strongly suggest that inhibition of mTORC1 activity in T cells upon rapamycin treatment decreases numbers of splenic effector T cells having the capacity to migrate into the brain (CD8+ CXCR3+) by day 6 of infection, a crucial step in ECM pathology [31–33]. Although the total number of activated CD69+ CD8+ T cells in the spleen was lower in the rapamycin treated group, the percentage of granzyme B+ effector T cells within this population was significantly increased, suggesting increased effector capacity. Finally, more CD62L+ naïve T cells were being directed to and retained in the spleen after rapamycin treatment. Together, this complex pattern of immune changes could contribute to increased clearance of parasitized RBCs in the spleen while at the same time reducing migration to the brain. Rapamycin also facilitates the generation of memory T cells in several models of infection and anti-tumor immune response [34, 35]. Future studies are required to determine if immunomodulation with rapamycin could have benefits on the memory T cell response in addition to affording acute protection against deleterious inflammation.

Vascular endothelial cell activation and dysfunction is another feature of severe disease upon malaria infection [20]. In vitro, mTORC1 inhibition decreases expression of adhesion molecules such as ICAM-1 and VCAM-1 at the surface of endothelial cells [22, 23]. In the context of ECM, a reduction of brain ICAM-1 protein expression was observed in the vasculature upon rapamycin treatment that could contribute to the reduction of parasite and leukocyte sequestration in this organ late in infection. However, as neurovascular activation in ECM is influenced by cytotoxic T cell migration to the brain, the relative cause and effect attributable to reduced adhesion molecule expression vs. immunomodulation will be difficult to disentangle using systemic rapamycin treatment.

While sequestration of parasitized RBCs in peripheral organs including WAT occurs both in human and rodent CM, the contribution of this process to neuropathology is unclear. It is expected that the failure to sequester will facilitate clearance of mature parasitized RBCs in the spleen, thus reducing parasite loads. In line with this, rapamycin treatment induced a significant decrease in parasite sequestration in both WAT and lungs without affecting circulating parasite levels, consistent with an increase in clearance by the spleen. Mechanistically, the data presented suggests both direct and indirect roles of mTORC1 in promoting sequestration. In specific tissues, this could be due to modulation of CD36 expression. Reduction of CD36 expression in WAT upon rapamycin treatment suggests a direct effect of mTORC1 in controlling cytoadherence via CD36 in this tissue. Conversely, CD36 was not differentially regulated in lungs. However, the fact that rapamycin significantly reduced parasite sequestration in lungs of infected mice suggests the existence of alternative mechanisms in addition to CD36 required to modulate parasite cytoadherence in this organ. Additional studies are required to elucidate such CD36-independent mechanisms of lung cytoadherence regulated by rapamycin.

The robust protection obtained upon a single dose of rapamycin late in infection justifies the evaluation of rapamycin analogs with improved pharmacological properties as potential therapeutic agents against ECM immunopathology. Torins, for instance, have effects against *Plasmodium* parasites; however, their effect as immunomodulators during ECM has not been assessed [15]. Interestingly, dihydroartemisinin, the active metabolite of all artemisinin-derived anti-malarials, has also been reported to inhibit mTORC1 in cells [36] and mice [37]. However, the time course of reduced mTORC1 activity is strongly suggestive of indirect effects mechanistically distinct from rapamycin. In addition, rapamycin has also been reported to have microbicidal activities against *Plasmodium* parasites [38]. However, the in vitro rapamycin concentrations necessary to affect parasite growth are much higher, suggesting this is not the mechanism underlying protection against ECM in most experimental settings [38].

Clinical testing and development of adjunctive therapies remain an urgent need due to the ongoing high fatality rates and long-term deleterious consequences of CM. In this context, novel potential adjunctive therapies with the ability to afford protection against ECM late in infection have been recently described. These include rosiglitazone and the glutamine analog 6-diazo-5-oxo-l-norleucine (DON) [8–10, 39]. Rosiglitazone, a direct PPAR-ɣ agonist, is effective in preventing death from ECM when given together with artesunate at days 5/6 post-infection [9, 39]. Rosiglitazone increases survival and phagocytosis of infected erythrocytes via upregulation of CD36 expression on macrophages [9, 39], and has proven safe in patients with uncomplicated *P. falciparum* malaria, thus prompting its use as an adjuvant therapy against CM [9, 40, 41].

DON, on the other hand, protects mice via inhibition of T cell degranulation, despite having no effect on accumulation of these cells in the brain when treatment was started on day 5 and continued every other day or every day. The pharmacokinetics and safety profiles of DON in humans will be crucial factors in establishing the appropriateness of the drug for the treatment of CM. In this context, a potential advantage of rapamycin is the ability to give a single effective dose, thus mitigating the potential side effects of continuous treatment.

## Conclusion

The data presented here demonstrate that rapamycin, a FDA-approved mTORC1 inhibitor, offers significant protection against ECM symptoms and mortality in mice even after a single dose administered late in infection via inhibition of multiple pathogenic mechanisms, and place rapamycin as a strong candidate for clinical use as an adjunctive therapy against human CM.
